# Light and Vision

**Published:** 1900-07-14

**Authors:** 


### LIGHT AND VISION.

While on the subject of the effect of light upon ordinary tissues it may be well to refer to the influence which it exerts upon the special structures in the retina and by which it produces those changes which lead to visual sensation. In the Bowman Lecture, recently delivered before the Ophthalmological Society, Mr. Marcus Gunn has shown that, on exposure to light, changes take place both in the pigment epithelium and in the rods and cones—the neural or visual epithelial cells—whose ends rest upon them. When stimulated by light the pigment particles contained in the pigment cells wander towards the light. Again, the pigment cell processes swell, and consequently grasp the outer segments of the visual epithelium; and finally the reaction of the contents of the pigment cell is altered, and it seems not impossible that this alteration varies in amount according to the wave-length of the light falling upon the cells. In the visual epithelium (1) the purple of the rods undergoes change of colour whenever light falls on the retina, and ultimately absolute bleaching takes place, the rapidity of the process depending upon the intensity of the light. (2) Movement takes place in the cones, and more feebly in the rods. While a cone protrudes well into the pigment when protected from light, it retracts rapidly on exposure, which would seem to be an illustration of direct movement being produced in excitable protoplasm by light. (3) There is a photo-electrical response, it having been conclusively demonstrated that the action of light upon the retina is accompanied by a marked electrical change. We have, then, in the eye an orderly utilisation of the energy of light, so as to set up that series of changes in nerve tissue which eventuate in visual sensation, while we can hardly doubt that some of the changes which are produced when light falls into the eye have the effect (perhaps we may say the object) of turning to useful, or at the least to innocuous purposes, those other and more destructive forms of energy, the heat and the actinism, with which light rays are so usually associated.

